# Design of Carryable Intravenous Drip Frame with Automatic Balancing

**DOI:** 10.3390/s20030793

**Published:** 2020-01-31

**Authors:** Ming-Feng Wu, Chia-Shan Chen, I-Shan Chen, Tz-Hau Kuo, Chih-Yu Wen, William A. Sethares

**Affiliations:** 1 Division of Chest Medicine, Department of Internal Medicine, Taichung Veterans General Hospital, Taichung 407, Taiwan; heriknoha@vghtc.gov.tw; 2 Department of Medical Laboratory Science and Biotechnology, Central Taiwan University of Science and Technology, Taichung 406, Taiwan; 3 Department of Electrical Engineering, Innovation and Development Center of Sustainable Agriculture (IDCSA), National Chung Hsing University, Taichung 402, Taiwan; wu165411@gmail.com (C.-S.C.); g106064095@mail.nchu.edu.tw (I.-S.C.); g107064020@mail.nchu.edu.tw (T.-H.K.); 4 Department of Electrical and Computer Engineering, University of Wisconsin-Madison, Madison, WI 53706, USA; sethares@wisc.edu

**Keywords:** carryable drip frame, balance control, sensor devices and systems

## Abstract

Due to the inconvenience of the conventional intravenous drip frame, the piggyback intravenous drip frame is developed to improve the mobility of the patient. However, the current design of the drip frame leads to a lack of balance control and increment of blood returning. To this end, the proposed system aims to solve this problem, and a fuzzy proportionalintegral–derivative control technique is developed to demonstrate the system feasibility. Accordingly, a reliable balanced system can be applied to facilitate patients’ movements and ensure patient safety with compensating the inclination angle of the drip frame such that the reduction of blood returning and the balance control of the piggyback intravenous drip frame can be achieved.

## 1. Introduction

Advances in the Internet of Things, wearable systems, data analysis, sensor devices, and medical applications may contribute to pervasive healthcare solutions and significantly improve the effectiveness, efficiency, safety, and sustainability of healthcare services [1,2,3]. This paper looks at sensor devices and systems of a drip frame design from the perspective of healthcare services for facilitating patients’ movements and ensuring patient safety in hospital environments. The drip frame is an essential tool for supporting the intravenous flow of liquids. The potential energy from the drip frame overcomes the venous pressure and places the drip into the IV site [4,5]. Currently, two types of frames are commonly used: one is arranged at the front end of a bed body, and the other is attached to a wheelchair. However, the body of a wheel-type drip frame is too long and has a considerable weight, which can impair mobility considerably. 

This paper describes a carryable and automatically balanced intravenous drip frame which allows the patient to move freely. Subjects wearing the new drip frame rise from bed, go to the toilet, eat, or walk up and down stairs, thus increasing the quality of life. The proposed system contains a carrying unit and a supporting unit where the supporting unit is a vest worn on the body and has a lightweight skeletal joint structure with a carrying unit on its shoulder for hanging the dripping frame. The carrier unit is provided with angle monitoring and compensation controlled using a fuzzy logic controller. The proposed system avoids the problem that the patients’ blood may flow back into the drip by suitable regulation of the pressure, and the system may be tilted forward or backward. In addition, it can be individually adjusted for different users.

**Figure 1:**
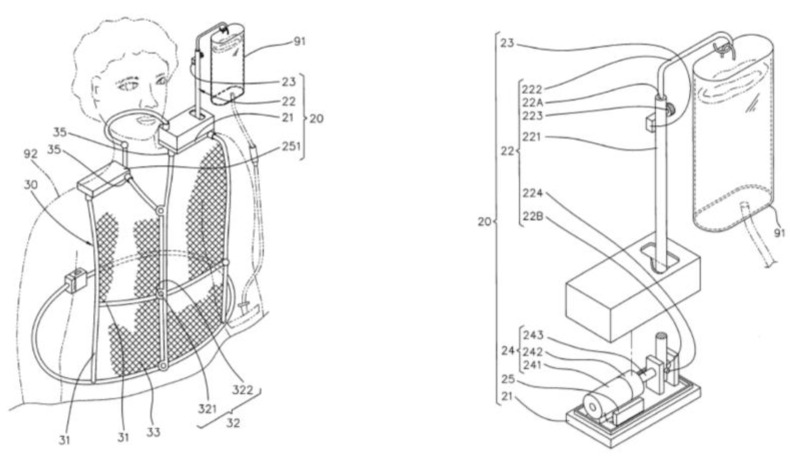
The conceptual system architecture [7].

In order to overcome the disadvantages of the conventional drip frame, this paper references the conceptual system architecture in our previous work [6] and develops a control strategy of the piggyback intravenous drip frame based on a motion sensor, a motor with proportional–integral–derivative (PID) control, and angle information, which uses the effect of attitude determination and dynamic response performance of a piggyback intravenous drip frame. By using a complementary filter with inertial navigation, the position of the drip frame can be corrected such that the infusion tube can be maintained at a certain height, which reduces the risk of blood return. Figure 1 shows the conceptual system architecture.

The key features of the proposed system are:

- The device can be moved with the human body
- The system can cooperate with shoulder activities and neck activities
- The control mechanism can correct the tilting angle of the body forward and automatically restore the angle

**Figure 2:**
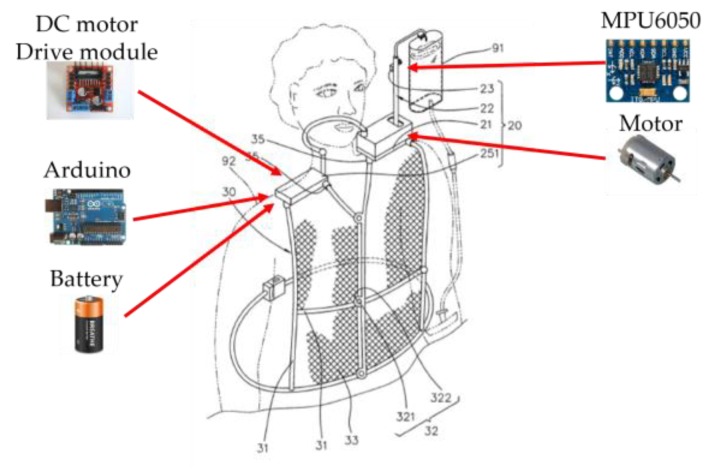
Carryable and automatically balanced intravenous drip frame.

**Figure 3:**
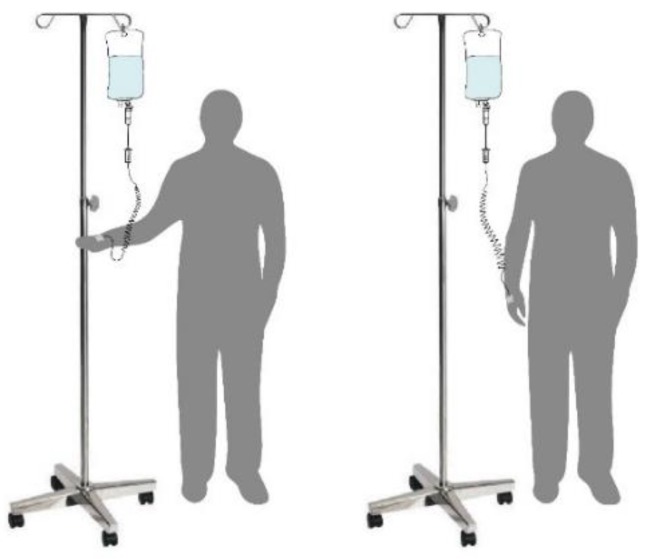
Traditional drip frame.

Thus, the proposed system is more convenient for patients to do activities (e.g., eating food, exercising, going to the toilet) in the hospital. As shown in Figure 2, compared with a portable ringer’s solution injection apparatus [8,9], the intravenous drip frame can control the drip frame to maintain a balanced state (perpendicular to the ground) despite body tilting, so the drip frame can be continuously maintained at an appropriate height. Therefore, the proposed system is more convenient than a conventional drip frame (Figure 3).

This paper is organized as follows: Section 1 reviews related work about the drip frame. Section 2 presents the design principles of the proposed system. Section 3 describes the implementation of the system. Section 4 evaluates the system performance and presents a performance comparison among the proposed control mechanism, the conventional PID scheme, the Ziegler–Nichols scheme [10], and the Tyreus-Luyben scheme [11]. Finally, Section 5 draws conclusions and shows future research directions.

## 2. Related Works

**Figure 4:**
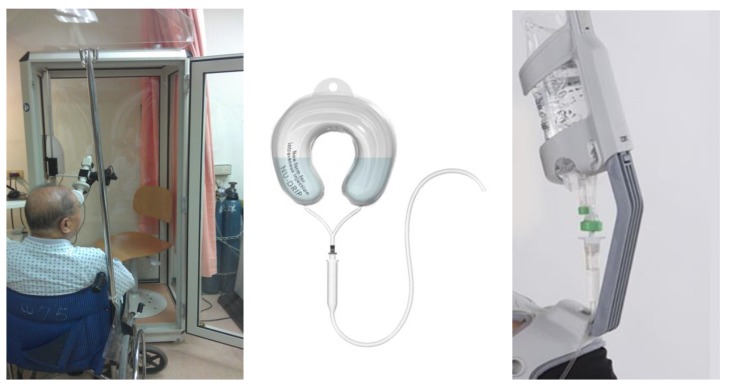
Examples of drip frames: a drip frame attached to a wheelchair (left); a new U-shape form for intravenous infection [12] (middle); a shoulder-mounted drip frame, EZPole, designed by Mobiu Corporation Ltd [16] (right).

Considering the conventional design of a drip frame, the fixed drip frame, including pulleys (Figure 4 (left)), lacks agility and convenience of movement. Therefore, several inventions are proposed to overcome this issue. As shown in Figure 4 (middle), a kind of wearable drip bag, called Nu-drip, is designed for intravenous therapies [12]. The U-shape pillow design can be worn on the neck comfortably, which lets patients move freely by themselves. Normal drip bags must be hung on a stand or held by others. Nu-drip’s design can reduce the risk of accidents caused by traditional drip bag stands, and it is safer for patients to move around with it in the hospital. However, this Nu-drip is not stabilized during patient movement, and the height between the dripping bag and the heart is not carefully considered, which may lead to the blood returning problem. 

In order to provide increased convenience of movement, the invention [13] relates to a shoulder-mounted multifunctional hanging device having a structure for fitting onto the body of a user, which consists of a support unit having a connection portion extending in the lateral direction, a concave portion contacting the acromion, a support unit mounted on the supraspinatus, and a recess unit. The shoulder-mounted multifunctional hanging device of the present invention is shaped to fit to the structure of the shoulder of a user, and the user may thus be administered an infusion solution in a more comfortable manner with less inconvenience to the shoulder.

**Figure 5:**
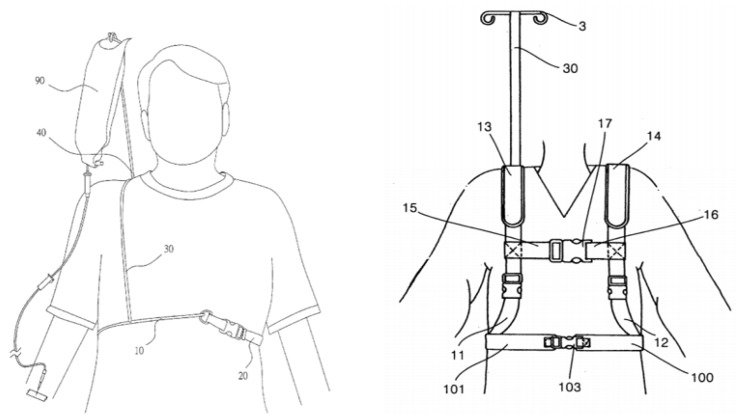
Taiwan Patents M404714 [13] (left) and M360702 [14] (right).

Although the new creative drip frame such as a shoulder-mounted (or mobile) drip frame (e.g., Taiwan Patents M404714 [14], M360702 [15], EZPole [16] as shown in Figure 4 (right) and Figure 5) allow for increased mobility, they cannot correct the position of the drip frame when the user leans forwards or backwards (or makes other actions). This implies that the drip frame will tilt with the user, thus reducing the height of the infusion tube and increasing the risk of blood return. Another kind of mobile design, such as the portable intravenous drip pressurization injection device (Taiwan patent M368452 [17]), uses additional pressure to drive the drip output. However, the device is mounted on the waist, and it may be difficult to observe the remaining amount of drip fluid.

Notice that the above designs of the piggyback intravenous drip frame lead to a lack of balance control and a possible increment of blood return. This work aims to solve this problem with PID control techniques and an inverted-pendulum system such that a reliable system may be applied to facilitate patient’s mobility and ensure the patient’s safety by compensating the inclination angle of the piggyback intravenous drip frame. Table 1 describes the comparison of drip frame technologies. 

## 3. System Description

**Figure 6:**
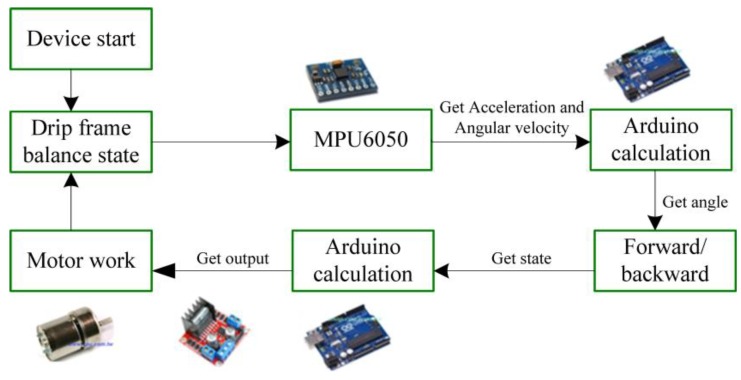
The conceptual system workflow.

In order to obtain information about the motion of the patient, a gyroscope is used to estimate the acceleration, angular velocity, and tilt angle. Complementary filtering then allows us to know whether the drip frame is tilted forward or backward and allows adjustments for the motor shaft. Moreover, by judging the weight of the drip bag from a weight sensor, PID parameters are adaptively modified by the weight of an object. Afterwards, the tilt angle is input into the PID calculation and the PID drives the motor. Finally, a positive and negative control to the piggyback intravenous drip frame through the motor, as shown in Figure 6, ensures that the height of the drip frame (32 cm from the heart) is properly maintained. The following subsections detail the design of the drip frame, the complementary filtering, and the PID controllers.

### 3.1. Drip Frame

Intravenous therapy uses the pressure generated by the height difference to regulate the flow of the liquid into the body. Therefore, it is important to maintain the drip at a certain height above the body in the system. Measuring the venous pressure and knowing the liquid potential, the height of the stent can be calculated, which means that the minimum liquid potential should be able to overcome the venous pressure [18]. Accordingly, given the general venous pressure 35 (cm H_2_O) = 35 (g/cm^2^), the liquid density 1.1 (g/cm^3^), and the height difference between the heart level and the bottom edge of the drip bag, h, the relationship between P and h yields.

P = ρh = 1.1(g/cm^3^) *h (cm) > 35 (g/cm^2^), (1)

which suggests h > 31.8 cm. Considering the patient’s mobility and safety, the height of the stent is set to 32 cm, which reduces the chance of blood returning.

### 3.2. Angle Calculation

To obtain the angular position of the object, both accelerometer and gyroscope data are applied. The gyroscope can achieve the above purpose by integrating the angular velocity over time. Moreover, in order to obtain the angular position with an accelerometer, the system determines the position of the gravity vector by using an arctangent function. Since the accelerometer data and gyroscope data are reliable over a long time interval and a short time interval, respectively, the data is further processed with a complementary filter.

#### 3.2.1. Acceleration

**Figure 7:**
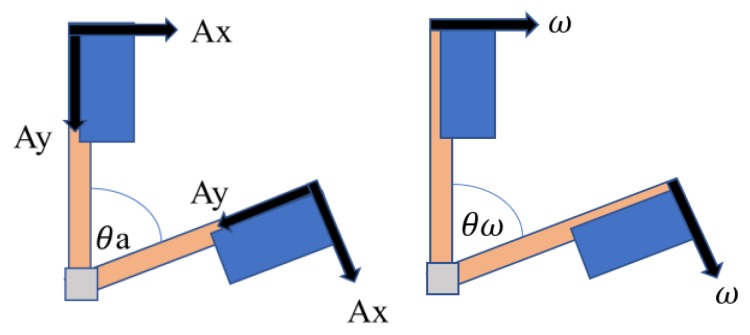
The acceleration angle (left); the angular velocity angle (right).

As an accelerometer measures all forces that are working on the object, it provides more information than that of the gravity vector. Note that every small force working on the object will disturb the measurement. If a system works on an actuated state (such as the self-balancing car), the forces that drive the system will be visible on the sensor as well. Since the accelerometer data are reliable only for a long period of time, a low-pass filter may be used. Let θ_ω_ and θa be the angles derived from gyroscope data and acceleration data, respectively. As depicted in Figure 7, acceleration angle θa is given by

(2) $$\theta a=\tan^{-1}\left(\frac{Ay}{\sqrt{Ax^{2}+Az^{2}}}\right)$$

where *Ax*, *Ay*, and *Az* are the X-axis, Y-axis, and Z-axis acceleration data, respectively.

#### 3.2.2. Angular Velocity

Due to the integration over time, the problem with gyroscopes is that the measurements of gyroscopes have a tendency to drift, not returning to zero when the system moves back to its original position. The gyroscope data are reliable only over short time periods. Hence, the angular velocity angle yields.

(3) θ[n]=θ[n−1] + ωdt

(4) $$\theta_{\omega }\;=\;\theta \left[n\right].$$

### 3.3. Complementary Filter

The complementary filter can be used to estimate the rotation angle (θ)

(5) $$\theta =K\times \theta_{\omega }+\left(1-K\right)\times \theta_{a}$$

where K is a constant. In this work, two types of complementary filters [19] are considered: (1) the traditional complimentary filter and (2) the adaptive complementary filter.

#### 3.3.1. Traditional Complementary Filter

**Figure 8:**
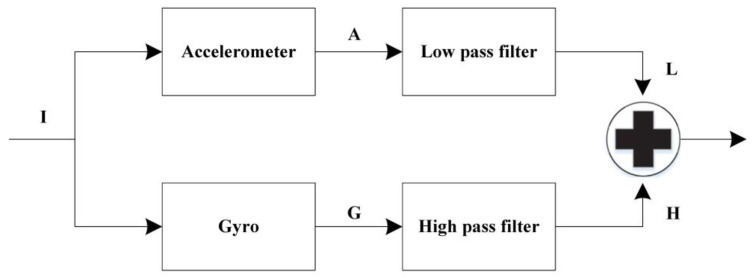
Conventional complementary filter.

Referring to Figure 8, the idea is to pass the accelerometer signals through a low-pass filter and the gyroscope signals through a high-pass filter, and then combine them to obtain the final rate. In the short term, the proposed system uses the data from the gyroscope, since they are precise and not susceptible to outside forces. In the long term, the proposed system uses the data from the accelerometer, as they do not drift. Note that the values of the complementary filter in Equation (6) are used for the ratio in which inertial sensor data are fused, which can then be applied to compensate for the measurement drift. The value for the angle derived from gyroscope data is commonly greater than or equal to 0.9 [20,21]. In this work, the value ω is set to 0.98. Accordingly, the filter equation is given by

(6) $$\mathrm{Angle}\;=\;\omega \cdot \theta_{\omega }\;+\;\left(1-\omega \right)\cdot \theta_{a}=\;0.98\cdot \theta_{\omega }\;+\;0.02\cdot \theta_{a},$$

where the gyroscope data are integrated with the low-pass data from the accelerometer (already processed with the arctangent function). As depicted in Equation (6), the proposed system updates the pitch and roll angles with the accelerometer data by taking 98% of the current value, and adding 2% of the angle information calculated by the accelerometer. This ensures that the measurement has a small drift and is very accurate on the short term. Denote θ[n] as the angle information, which yields the equation below.

(7) $$\theta [n]\;=\;[K\;(\theta [n-1]\;+\;\omega \mathrm{dt})\;+\;(1-K)\;\times \;(\tan^{-1}(\frac{Ay}{\sqrt{Ax^{2}+Az^{2}}}))].$$

#### 3.3.2. Adaptive Complementary Filter

Referring to Equation (5), as the system oscillates, the angle information is highly based on the angular velocity angle, which implies that the value of K needs to be increased to meet the system demand. In contrast, when the system is stable, the value of parameter K needs to be reduced in order to achieve the convergence of the angular velocity error. Let K[n] be the discrete-time signal of K, and denote the difference between angular velocity (w) and acceleration angle (a) as Z, which is

(8) Z=|w−a|.

**Figure 9:**
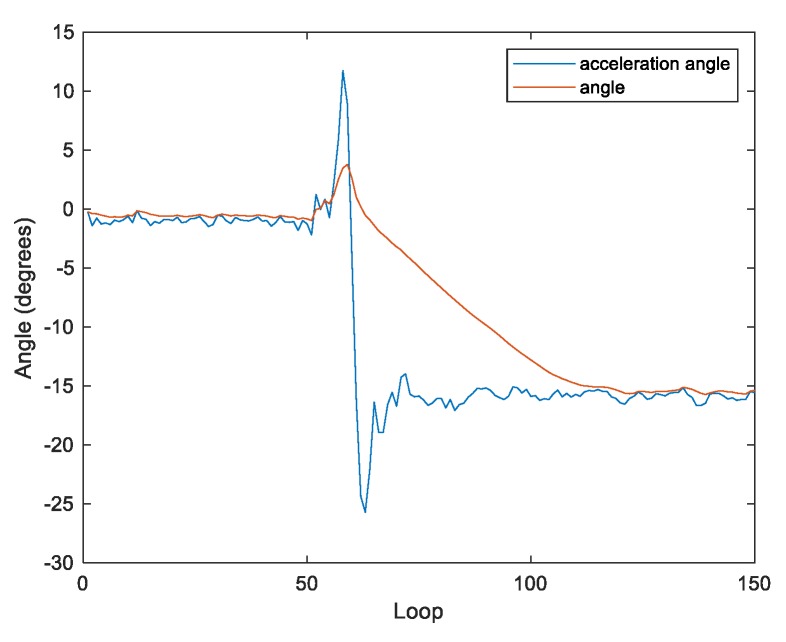
The reliability of the acceleration with R = 0.75.

**Figure 10:**
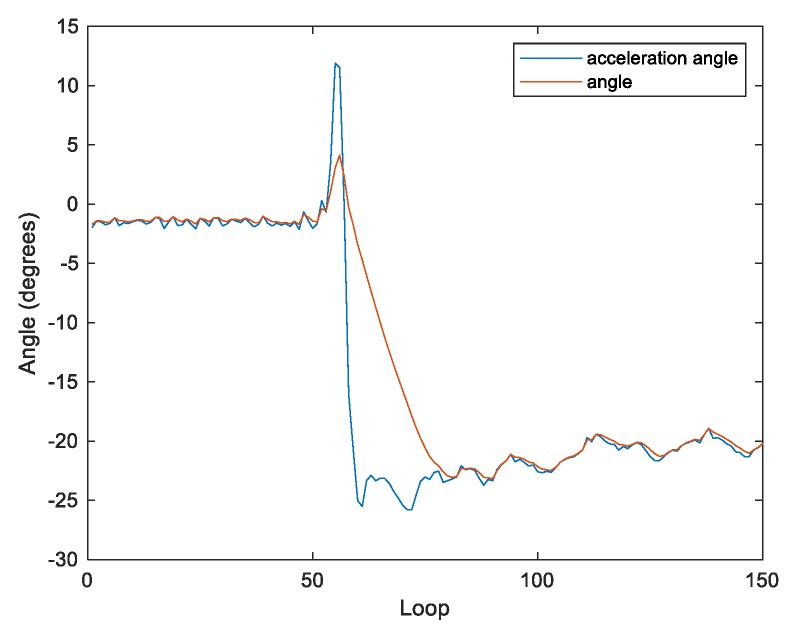
The reliability of the acceleration with R = 2.

In order to make K[n] a changing value with two inputs, the parameter R is applied to describe the reliability of the acceleration. As shown in Figure 9 and Figure 10, R is the difference between the angular velocity (w) and acceleration angle (a) when the system is stable, following from Equation (9). Thus, θ[n] can be rewritten as follows:

(9) K[n]=Z/(Z+R),

(10) $$\theta [n]\;=\;[K[n]\;(\theta [n-1]\;+\;\omega \mathrm{dt})+(1-K[n])\;(\tan^{-1}(\frac{Ay}{\sqrt{Ax^{2}+Az^{2}}}))].$$

**Figure 11:**
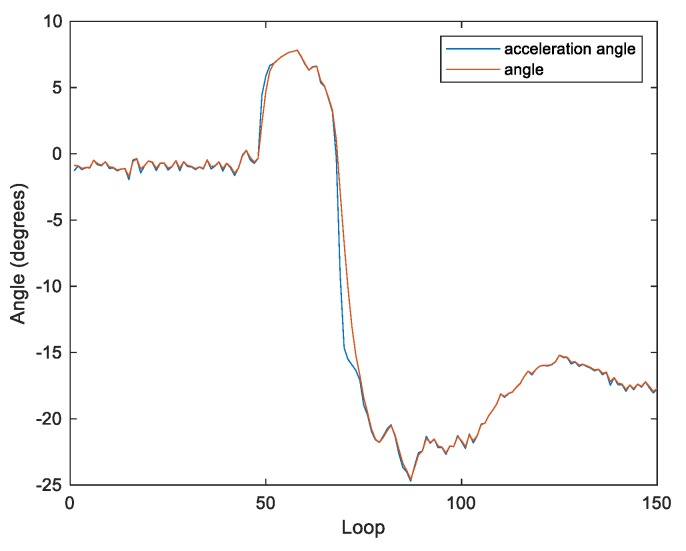
The reliability of the acceleration with R = 5.

The performance of the filter can be adjusted by tuning the parameter R. When R is less than 1, the filter is effective in reducing oscillations for achieving the steady state. However, the filter’s reaction time is too long to maintain the system balance when the body tilts (Figure 9). In contrast, when R is greater than 5 (Figure 11), the filter can calculate the tilt of the body faster. However, in the steady state, the output of the filter is too close to the acceleration, which is not effective in reducing oscillations. Therefore, in this work, R is set to 2 (Figure 10).

### 3.4. Conventional PID Controller

A proportional–integral–derivative (PID) controller is a control loop feedback mechanism that is widely used in industrial control systems and a variety of other applications with continuously modulated control. This section describes the three parameters—P, I, and D—of the PID controller. Term P is proportional to the current value of the tilt angle. Term I accounts for the past values of the tilt angle and integrates them over time to generate the I term. Term D is a best estimate of the future trend of the tilt angle, based on its current rate of change.

#### 3.4.1. PID Controller

**Figure 12:**
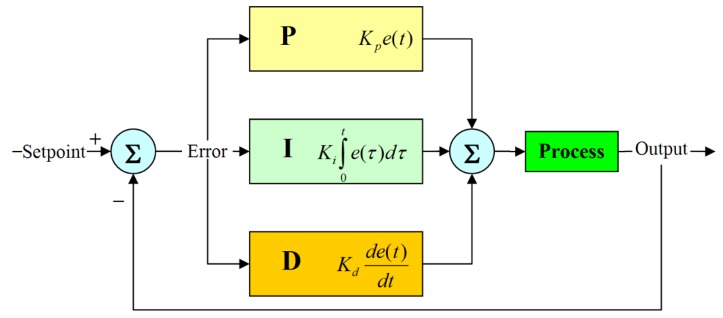
A block diagram of a proportional–integral–derivative (PID) controller in a feedback loop.

This controller compared the collected data with a set point, which is used to calculate the new input value for approaching the set point [22]. Since the key features of the PID controller are using a weighted sum of the control terms and considering the impacts of the three control terms (i.e., proportional, integral, and derivative influences) on the controller output, this system is able to minimize the tilt angle value over time by adjusting the PID output. A block diagram of a PID controller [23] is shown in Figure 12.

Note that K_p_, K_i_, and K_d_ are proportional, integral, and derivative gains, respectively. e(*t*) is the tracking error defined as the tilt angle. τ is the variable of integration, taking on values from time 0 to the present time *t*. The PID controller can also be expressed as follows:

(11) $$\mathrm{Output}=K_{p}e\left(t\right)+K_{i}\int_{0}^{t}e\left(\tau \right)d\tau +K_{d}\frac{de\left(t\right)}{dt},$$

which is the actual system output and can be described by the control effort at time *t* [24].

#### 3.4.2. The Ziegler–Nichols Method

Based on the above design concept, Ziegler–Nichols [10] presented a tuning function, considering the time response and empirical results. Although lacking parameter selections and having overshoots in time response, parameter adjustments are still achievable. The Ziegler–Nichols method is performed by setting the integral and derivative parameters to zero. The proportional parameter K_p_ then increases until it reaches the ultimate gain K_u_, at which the output of the control loop has stable and constant oscillations. Ku and the oscillation cycle Pu are used to set the P, I, and D parameters, depending on the type of controller used. Table 2 summarizes the controller parameters for the closed loop Ziegler–Nichols method.

Note that K_u_ is the proportional gain, τ_i_ is the integral time, τ_d_ is the derivative time with Ki = Kp/τ_i_, and Kd = Kp*τ_d_. Therefore, the values of Kp, Ki, and Kd are obtained from Table 2 to form the output function of the PID controller, as depicted in Equation (11).

#### 3.4.3. Tyreus–Luyben Method

The Tyreus–Luyben [11] procedure is similar to the Ziegler–Nichols method, but the final controller settings are different. Moreover, this method only proposes a method for PI and PID controllers. Table 3 presents the controller parameters for the Tyreus–Luyben method. Similar to the Ziegler–Nichols method, this method is time consuming for keeping the system stable and approaching the set point.

### 3.5. PID Controller Using Fuzzy Logic

**Figure 13:**
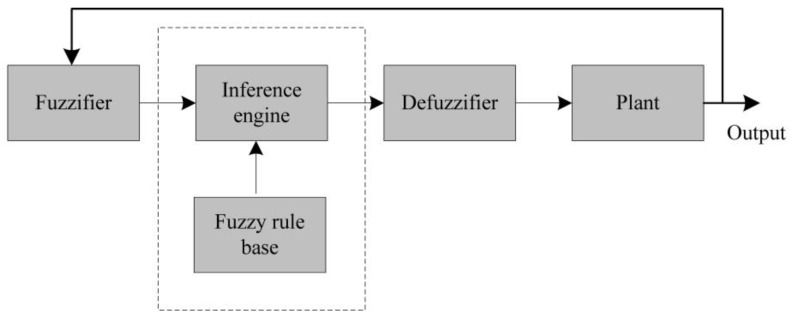
A typical architecture of fuzzy logic control.

Observe that the conventional controllers are not able to effectively reflect the variation of the weight of the drip bag and the tilt angle of the frame. Hence, we propose a controller with the concepts of fuzzy sets, linguistic variables, and approximate reasoning. Figure 13 shows the operation procedures, which comprises four principal components: fuzzifier, fuzzy rule base, inference engine, and defuzzifier [25].

#### 3.5.1. Fuzzification

**Figure 14:**
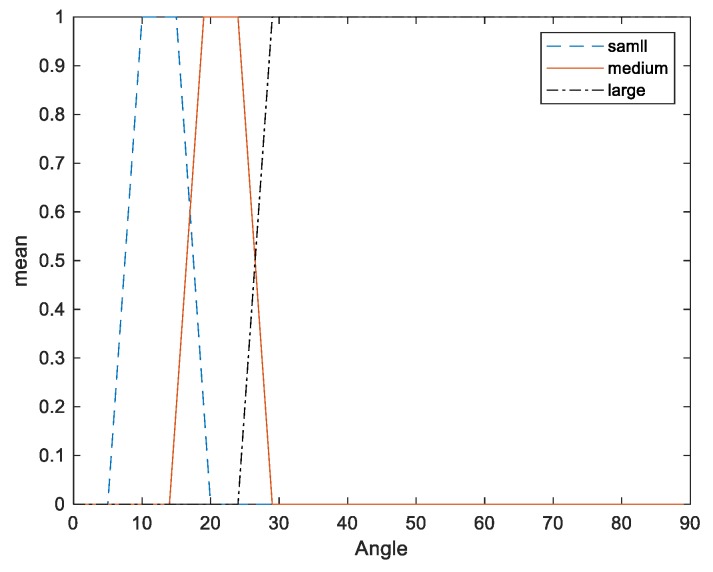
Membership functions of angle.

**Figure 15:**
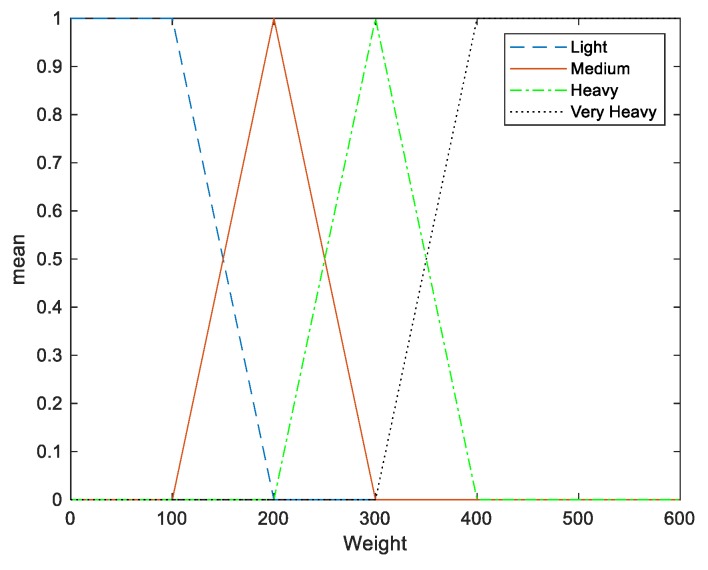
Membership functions of weight.

The fuzzifier has the effect of transforming crisp measured data (e.g., the weight is 150 g) into suitable linguistic values (i.e., fuzzy sets, for example, where the weight is moderate). Two inputs are considered in this fuzzy PID system: “Angle” and “Weight”. The input angle has three membership functions (Figure 14) and the weight has four membership functions (Figure 15). 

#### 3.5.2. Fuzzy Inference

**Figure 16:**
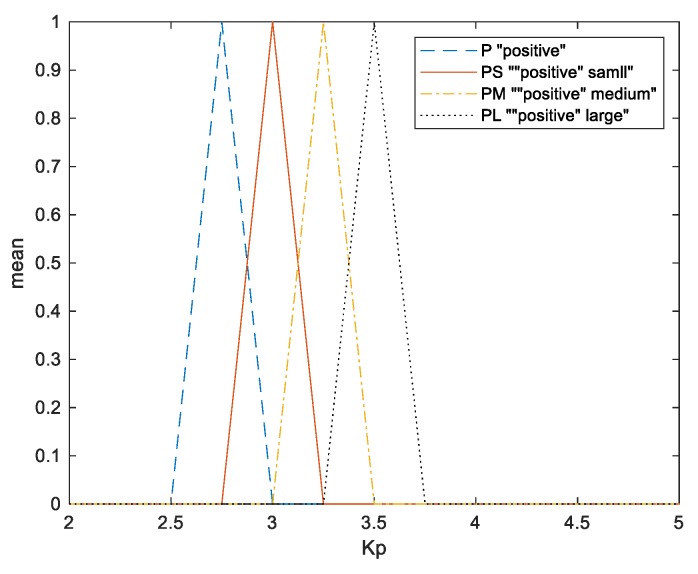
Membership functions of parameter Kp.

**Figure 17:**
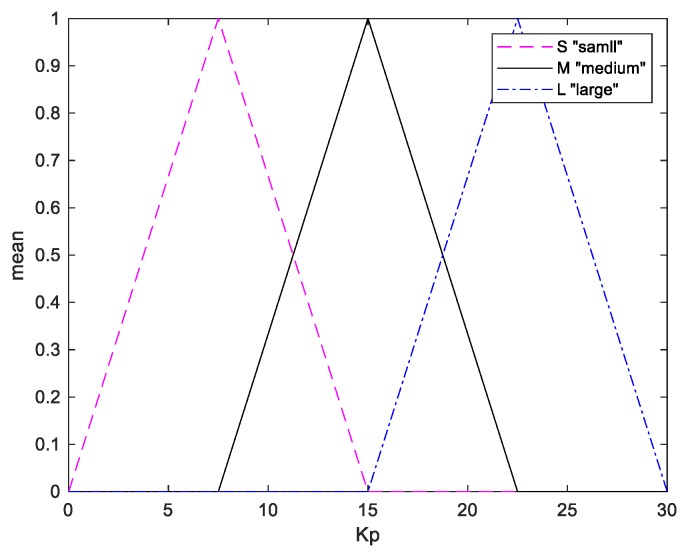
Membership functions of parameter Ki.

Numerous fuzzy conditional statements constitute a fuzzy rule base. As shown in Table 4, 12 rules are applied to adaptively adjust the Kp and Ki values with the weight and the angle information. The membership functions of Kp and Ki are shown in Figure 16 and Figure 17, respectively.

#### 3.5.3. Defuzzification

The defuzzifier is utilized to yield a non-fuzzy decision or control actions from an inferred fuzzy control action by the inference engine with the center of area (COA) method,

(12) $$Z_{COA}^{*}=(\sum_{j=1}^{N}\mu_{c}\left(z_{j}\right)z_{j})/\sum_{j=1}^{N}\mu_{c}\left(z_{j}\right).\;$$

**Figure 18:**
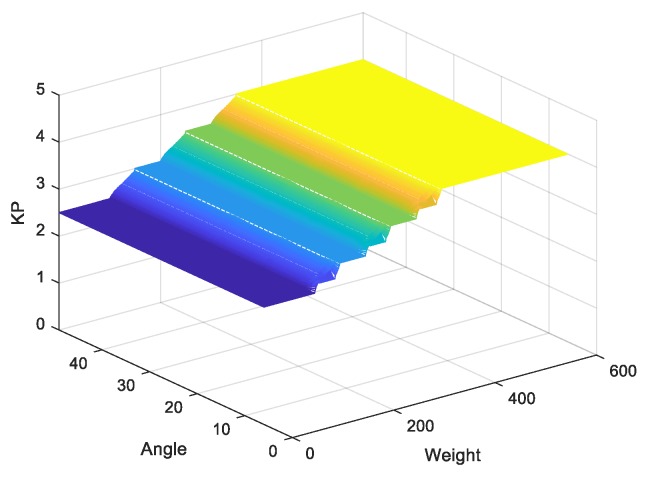
Output of Kp.

**Figure 19:**
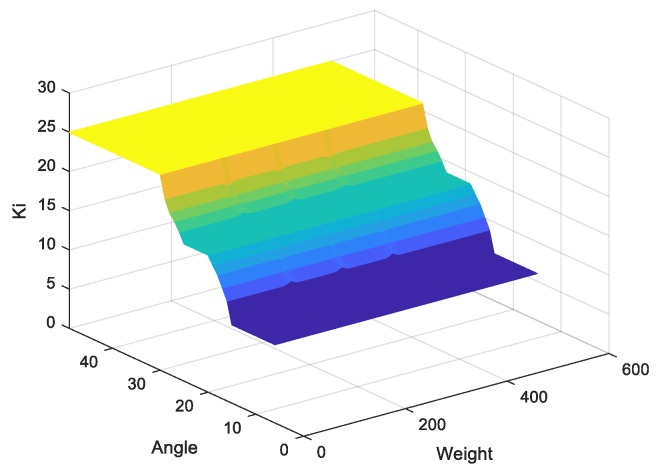
Output of Ki.

Note that $z_{j}$ is the amount of control output at the quantization level j, and $\mu_{c}\left(z_{j}\right)$ represents its membership value in the output fuzzy set Kp and Ki. Referring to angle and weight information, the outputs of Kp and Ki are described in Figure 18 and Figure 19, respectively.

### 3.6. System Workflow

**Figure 20:**
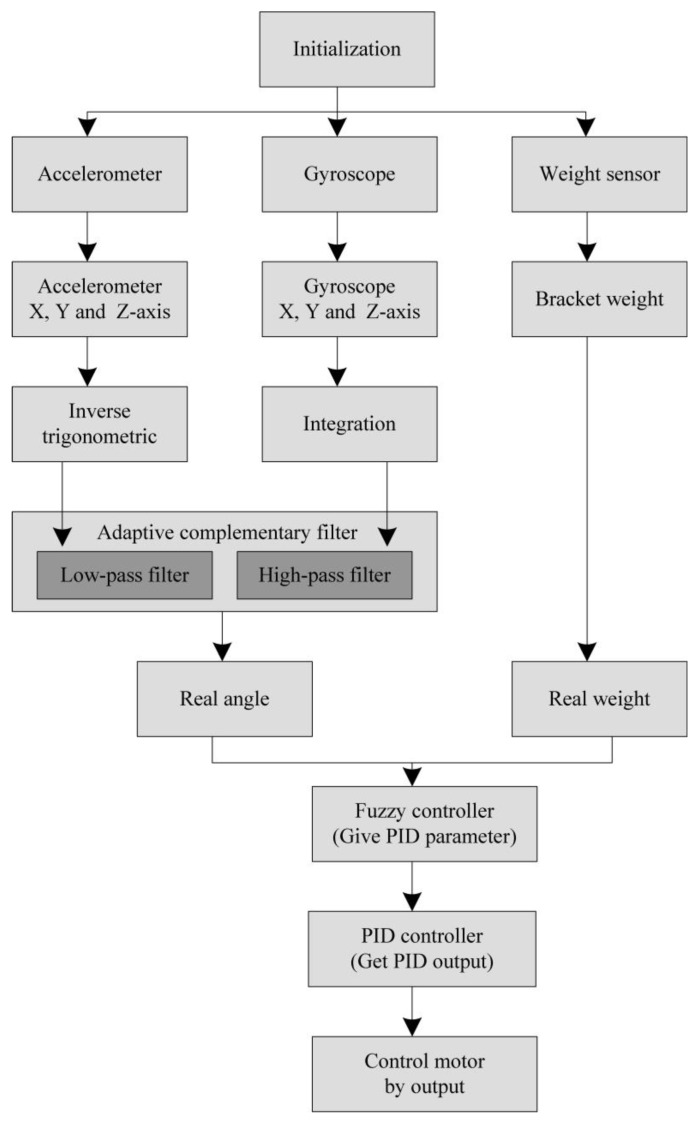
System workflow.

**Figure 21:**
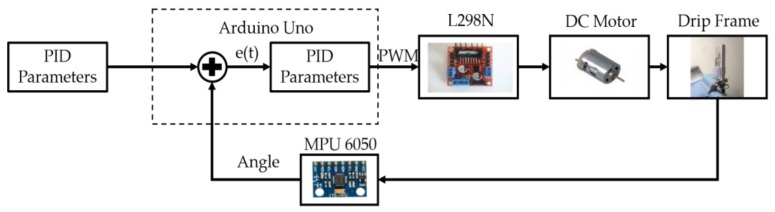
Closed-loop DC motor speed control scheme on PID controller.

The system workflow includes system initialization, data acquisition, data filtering, and data processing, as shown in Figure 20. After system initialization, the accelerometer and gyroscope data of the current state are collected. Using the operations of adaptive complementary filter to measure the derivative of the tilt angle and the combination of fuzzy PID control, the output of pulse-width modulation and the speed direction of the motor can be adjusted, which automatically compensates for the speed. Note that the angle data and speed data are filtered by the complementary filter to remove the abnormal data. Based on the measured angle and weight information, the proposed fuzzy controller is performed to balance the drip frame. Figure 21 shows a closed-loop DC motor speed control scheme. The equipment includes an Arduino UNO, L298N H-Bridge, DC power supply, DC motor, MPU6050, HX711 electronic scales, a braking system, and a computer with software and hardware.

### 3.7. System Prototype

**Figure 22:**
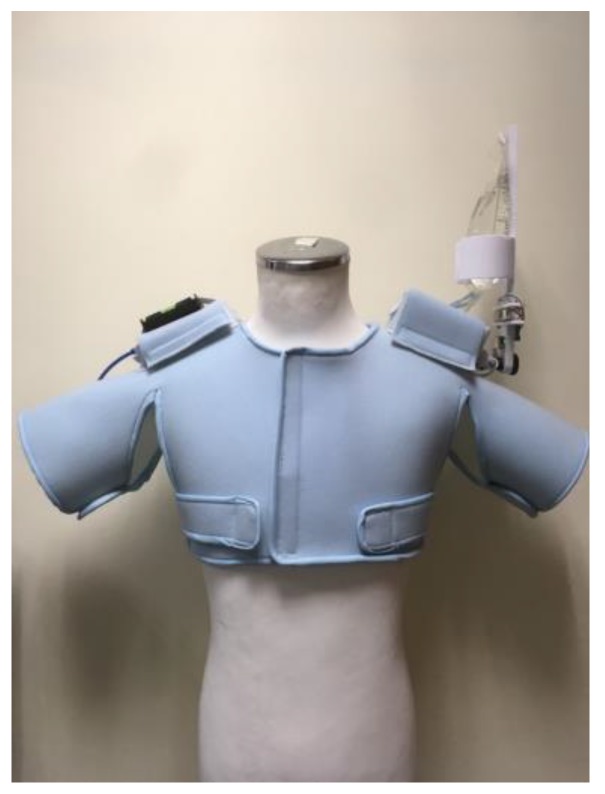
System prototype.

Figure 22 illustrates the system prototype, which consists of three parts: (1) a system hardware design, (2) a control scheme for the movement of the drip frame, and (3) a support vest. The system control frame has a structure related to the tilt angle (forward or backward angle), which includes a master control module, an angle sensor, and a DC motor. The range of the tilt angle change can be from −45 to +45 degrees for an angle of tilt. Note that a vest, made by polyester sandwich fabric with nylon support for producing a breathable structure, is designed for carrying the hardware and supporting the balanced drip frame. The target subjects are the patients with good consciousness and good shoulder strength.

The weight of the system on the left shoulder (including the drip bag) and that on the right shoulder are about 700 g and 200 g, respectively. Therefore, the load on the shoulder is less than 1 kg. Moreover, the length of the container is about 9 cm; the distance between the external ear and the drip frame is about 10 cm; and the distance between the neck and the container is about 5 cm. 

Compared with the average price of the conventional drip frame (60 US dollars), the total expense of the system prototype is about 62 US dollars, including the support vest, Arduino UNO, L298N H-Bridge, DC power supply, DC motor, MPU6050, and HX711 electronic scales. Therefore, the proposed system is cost-competitive with the conventional one. In order to assess the system feasibility, a questionnaire survey was conducted on patients who had ever used a conventional drip frame from April 2018 to May 2018 in Taichung Veterans General Hospital, Taiwan. A total of 30 patients aged from 23 to 91 were enrolled in the survey. The result shows that the complaints percentages about going to the bathroom, physical examination, and eating were 96.7%, 36.7%, and 26.75%, respectively. Results also shows that 90.0% respondents are willing to adopt the system prototype if the total weight of the system is less than 1 kg. As a result, the proposed carryable intravenous drip frame has a potential benefit to provide better life quality for a patient with intravenous injection. Referring to the design concept in Figure 2, the following subsections detail the system on the right shoulder and that on the left shoulder, respectively.

### 3.8. System on the Right Shoulder

**Figure 23:**
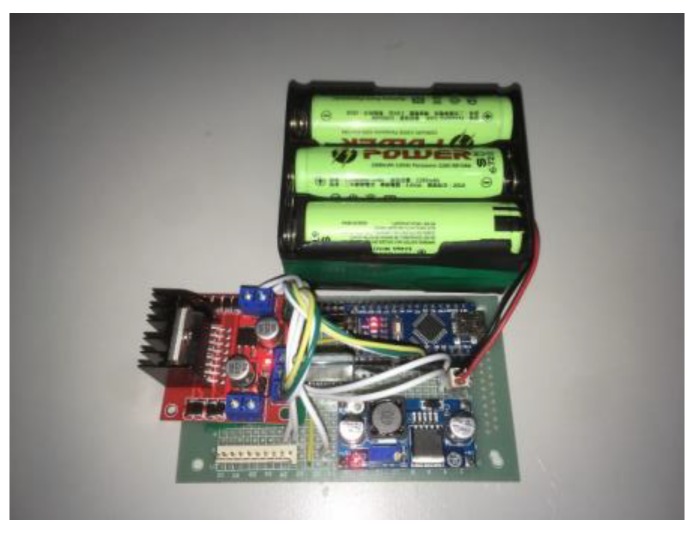
The circuit design of the system module.

This right-shoulder system module includes an Arduino UNO, L298N H-Bridge, DC power supply, and 24V and 9V battery modules. In the master control module, built-in AD digital-to-analog converters are used for data acquisition and processing, angle conversion, and motor control. Note that the L298N is a dual H-Bridge motor driver, which can drive and control two DC motors. Figure 23 shows the circuit design of the right-shoulder system module.

### 3.9. System on the Left Shoulder

**Figure 24:**
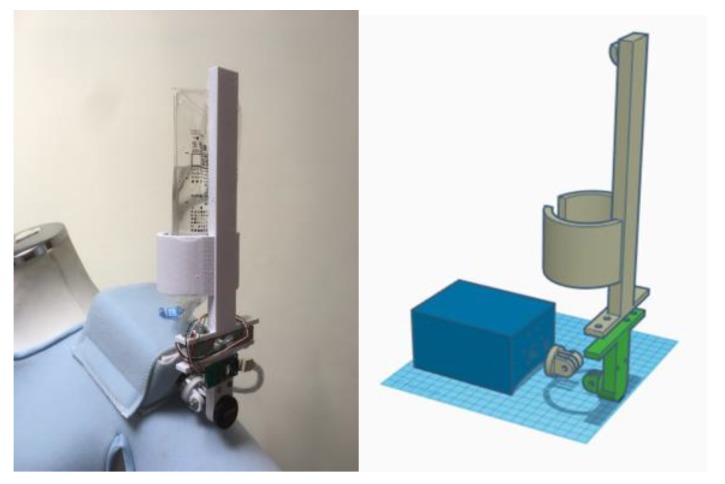
The left-shoulder module.

This left-shoulder system module includes the DC motor, MPU6050, HX711 Electronic Scales, and drip frame module (Figure 24). The MPU6050 module built-in gyroscope and accelerometer are used for sensor data acquisition and angle conversion. The built-in HX711 Electronic Scales module is applied to get the weight data for balancing the DC motor-control drip frame.

#### 3.9.1. MPU6050

**Figure 25:**
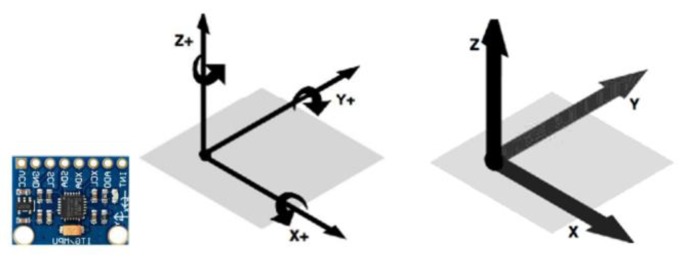
Accelerometer axis and accelerometer axis of MPU6050.

MPU6050 (module GY521 for Arduino) has a three-axis accelerometer and a three-axis gyroscope sensor, measuring the angular rotation and accelerations relative values for motion measurements (Figure 25). Improved measurement results may be obtained if a three-axis magnetometer is added in the measurement systems. The MPU6050 gyroscope has a scale range of ±250/s, ±500/s, ±1000/s, and ±2000/s [26]. It increases the bias, sensitivity, temperature, and stability, which may reduce the need for user adjustment, being able to program a digital low-pass filter for obtaining more accurate data measurements [27].

#### 3.9.2. HX711 Electronic Scales

**Figure 26:**
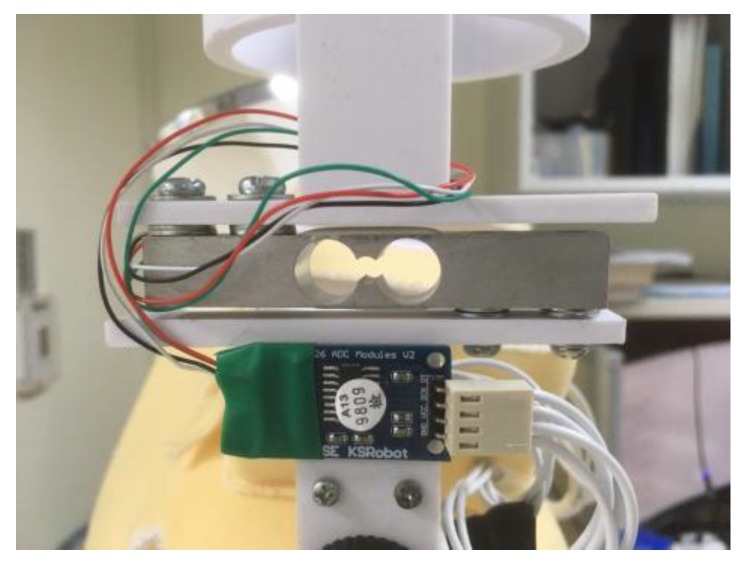
HX711 Electronic Scales.

**Figure 27:**
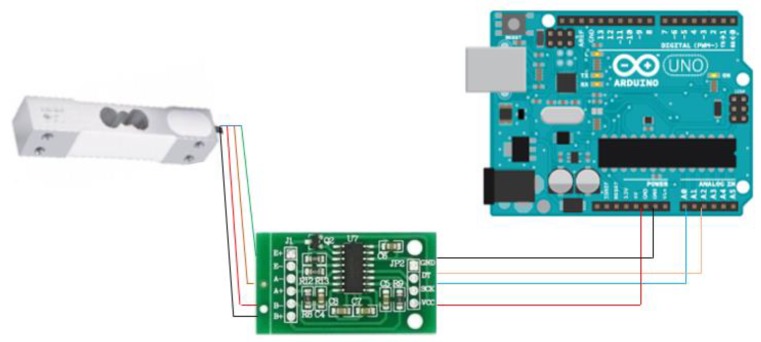
HX711 system: load cell (left); HX711 chip (middle); Arduino Nano (right).

As shown in Figure 26, HX711 [28] is a high-precision electronic scale with a 24-bit A/D converter chip from Rantle East Electronic, which is integrated with a regulated power supply and is located on a chip clock oscillator, which has the advantages of a high degree of integration, response speed, anti-interference ability, low cost, and high reliability. Then, the weight reading is transmitted to the controller through the amplifier of the HX711 chip for providing an accurate change in the resistance value. Figure 27 illustrates the key components of the HX711 Electronic Scales.

#### 3.9.3. DC Motor

This system uses a DC motor to control the movement of the drip frame, which is widely applied in high-precision servo systems because of their efficiency, high reliability, and easy maintenance for speed adjustment and the excellent control characteristics [29]. In this work, the motor model is 37GB520. It is mounted in the container on the left shoulder. Table 5 shows the motor characteristics. 

### 3.10. Android Application

**Figure 28:**
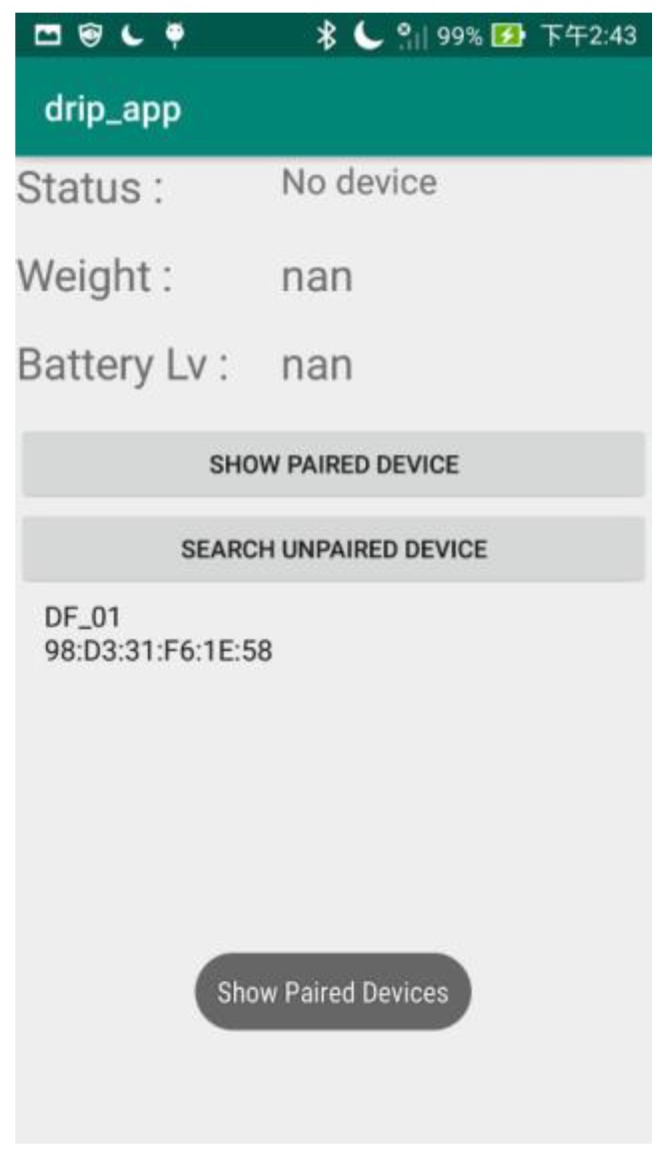
Android phone app application.

Android Studio provides the fastest tools for building apps on every type of Android device. In this system, the drip frame information may be transmitted to the user’s mobile phone through the Bluetooth connection for monitoring the status of the drip frame. Figure 28 is the start page of the app. The interface content includes the Bluetooth status, two text views to show the weight and battery status information, and two device pairing buttons. Moreover, the app can integrate with irregular error control settings, which may be described in terms of system components and their interactions (e.g., battery level, the sudden large change of the tilt angle). For the battery issue, the charge and capacity level of a battery can be monitored via an Arduino-based battery level sensor. For the issue of a sudden large change of the tilt angle (e.g., patient falls), a phone app can be used to make a call to the hospital calling center or the nursing station for the emergency assistance. Moreover, these settings can be further integrated with some system information (e.g., sending a message when the weight of the drip bag is approaching zero) to extend applications.

### 3.11. Communication Architecture

**Figure 29:**
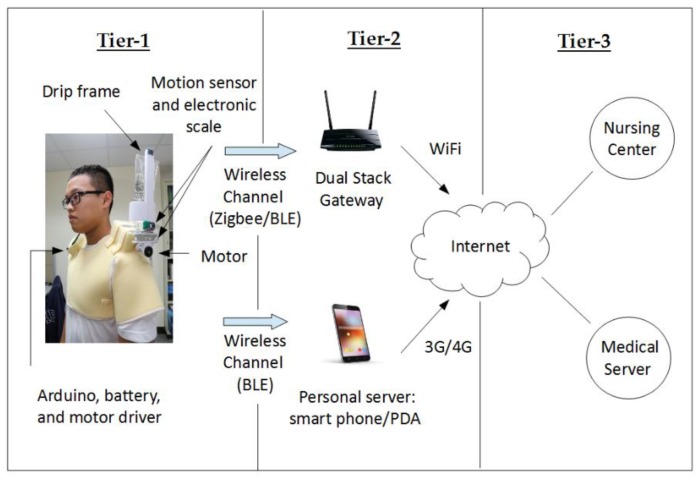
A three-tier communication architecture.

Based on system operations, Figure 29 describes the three-tier communication architecture of the proposed system. In Tier 1, the Arduino UNO collects the sensing information from a motion sensor and an electronic scale for the motor driver to balance the drip frame. In Tier 2, a personal server (e.g., smart phone/PDA) or a dual stack gateway communicates with the Arduino UNO via a wireless channel. For the proposed system, Bluetooth Low Energy (BLE) technology is applied to establish a communication channel between the Arduino UNO and the smart phone. In Tier 3, the Internet is used to establish a channel between a personal server and a medical server/nursing center. Therefore, in this work, a three-tier communication architecture is achieved with the drip frame with automatic balancing, a smart phone, and a medical database (or the nursing center). For the connection between Tier 1 and Tier 2 (i.e., the drip frame and a smart phone), the BLE technology is used. For the connection between Tier 2 and Tier 3 (i.e., a smart phone and a medical database (or the nursing center)), the Internet technologies are used. 

## 4. Results

To assess the system performance, two sets of experiments of a PID speed controller are conducted, considering the conventional PID method, the Ziegler–Nichols method, the Tyreus–Luyben method, and the proposed fuzzy PID method. These methods are compared on the basis of the output response, minimum settling time, and minimum overshoot for the speed demand application of the DC motor. Note that in order to avoid over-frequent adjustments of the tile angle and maintain the comfort of the patient, the blind zone of the tilt angle is set to 5 degrees. 

Two sets of experiments are applied to extract system behaviors. Referring to the system parameter settings in Table 6, the values for the Kp, Ki, and Kd parameters of the non-fuzzy controllers are the following: for the PID method, Kp = 5.4, Ki = 21.6, and Kd = 0.33; for the Ziegler–Nichols method, Kp = 2.97, Ki = 11.88, and Kd = 0.18; for the Tyreus–Luyben method, Kp = 2.81, Ki = 12.36, and Kd = 0.22. 

### Performance Comparison

The performance comparison between these methods is executed in terms of overshoot, settling time, and steady-state error. Note that the percentage overshoot/undershoot (PO/PU), settling time (ST), and steady-state error are calculated from the data through the convergence of the drip frame. The definitions of the percentage overshoot/undershoot, settling time, and steady-state error are as follows.

- *Percentage Overshoot/Undershoot*: Referring to the ratio of the value of angle overshoot/undershoot to the maximum angle deviation, with respect to the set point:
  $$\left|\frac{\min Dvi}{\max Dvi}\right|\times 100\%,$$
  where the set point is the tilt angle of zero degrees, **min *Dvi*** is defined as the value of the overshoot/undershoot, and **max *Dvi*** is defined as the absolute value of the tilt angle.
- *Settling Time*: The time required for balancing the system with a tilt angle, which deviates from the set point less than 5°.
- *Steady-State Error*: The difference between the set point and the average tilt angle after system stabilization.

**Figure 30:**
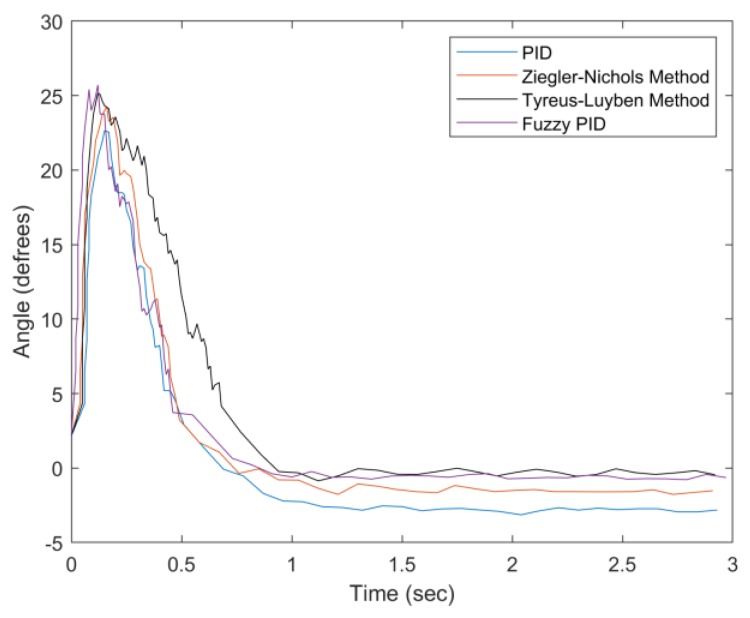
Convergence of angle with weight = 0 g.

Without loss of generality, assume that a user tilts forward. Thus, the percentage undershoot (PU) is considered. In the first set of experiments, given the tilt angle (about 25°), system performance is evaluated with varying the weight from 0 to 200 g. Figure 30 presents the angle adjustment with zero-gram weight. Compared with conventional methods, the proposed fuzzy PID method has a compatible settling time, overshoot suppression, and steady-state error. Observe that the Tyreus–Luyben method has the largest settling time and a smaller steady-state error. The PID method has the largest steady-state error due to the parameter settings.

**Figure 31:**
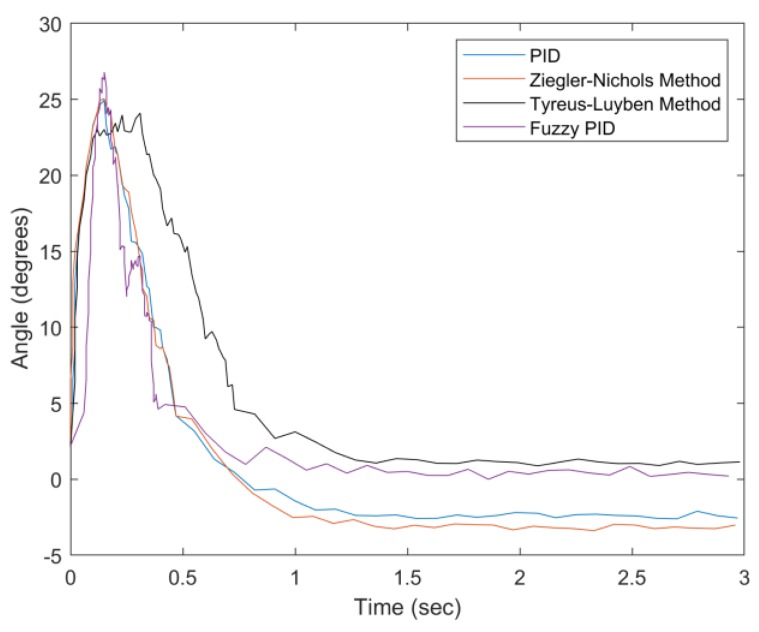
Convergence of angle with weight = 100 g.

Figure 31 describes angle adjustment with a 100 g weight, which indicates that the proposed fuzzy PID method has a superior performance in settling time, overshoot suppression, and steady-state error. As can be seen in Figure 31, the settling time of the Tyreus–Luyben method (about 0.73 s) is longer than that of the proposed fuzzy PID method (about 0.5 s), reflecting the variation of the weight of the drip bag. Note that besides the control mechanism, the support vest provides a stable base for the container. Therefore, the effect of the settling can be almost neglected by the user whether sitting, walking, or standing. In this work, the definition of settling time is the required time for balancing the system within the blind zone of the tilt angle (less than 5 degrees). Since the settling time is related to several factors, (e.g., the motor characteristics and the range of the blind zone), in the future, we will carefully explore these related factors to further optimize the system performance.

**Figure 32:**
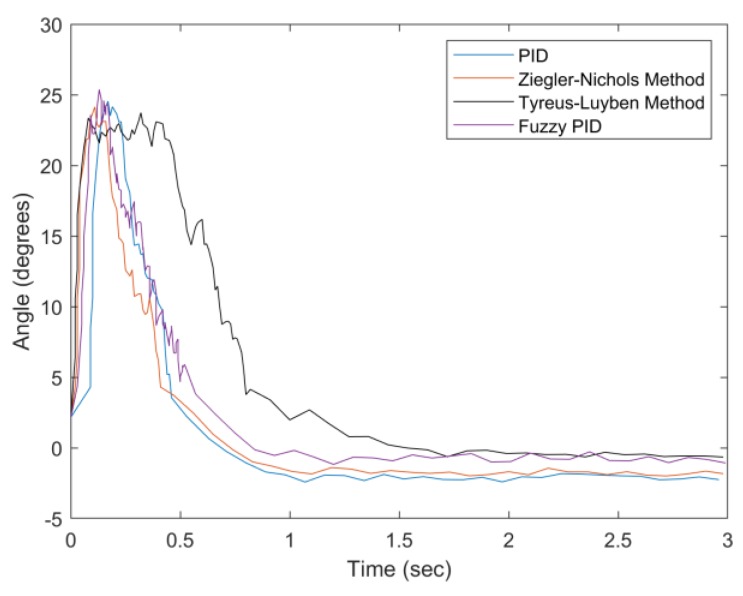
Convergence of angle with weight = 150 g.

**Figure 33:**
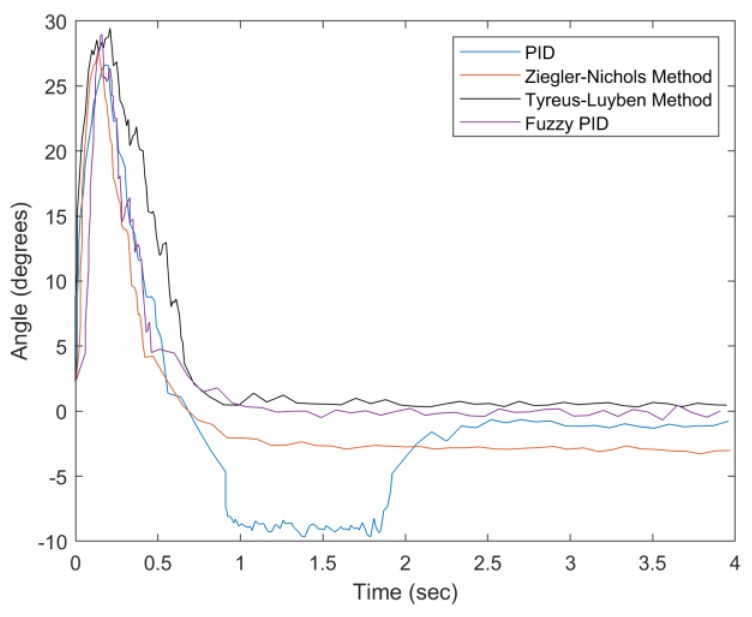
Convergence of angle with weight = 200 g.

In Figure 32, as the weight increases to 150 g, the Tyreus–Luyben method has the largest settling time (about 1.5 s). Considering the weight information for the system parameter settings, the proposed fuzzy PID method has a good convergent speed and the smallest steady-state error. Figure 33 highlights the performance differences with a 200 g weight. In this case, the PID and Ziegler–Nichols methods have larger settling times, undershoots, and steady-state errors. Moreover, the PID has the largest percentage undershoot, which indicates that it has excessive feedback due to the increasing weight. Table 7 and Table 8 summarize the experimental results of the first experiment. Observe that the proposed fuzzy PID method performs better than the other examined methods in various weight conditions in terms of settling time, percentage undershoot, and steady-state error.

**Figure 34:**
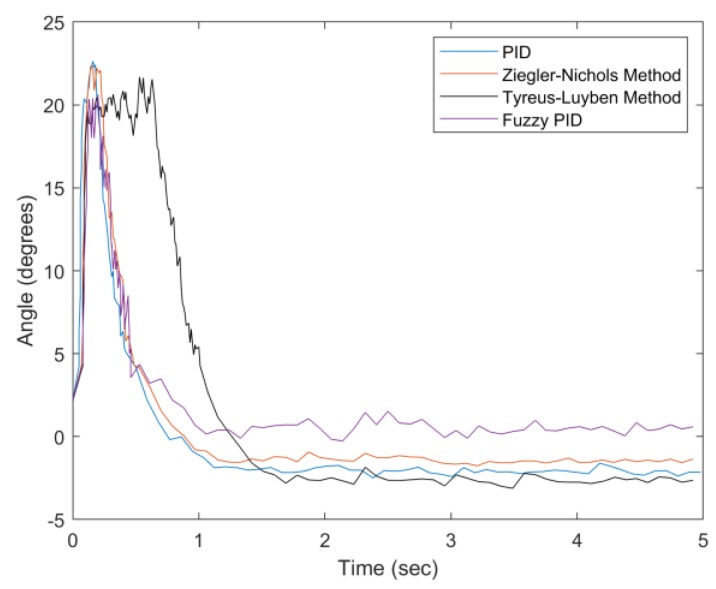
Tilt angle is approximately 20 degrees.

**Figure 35:**
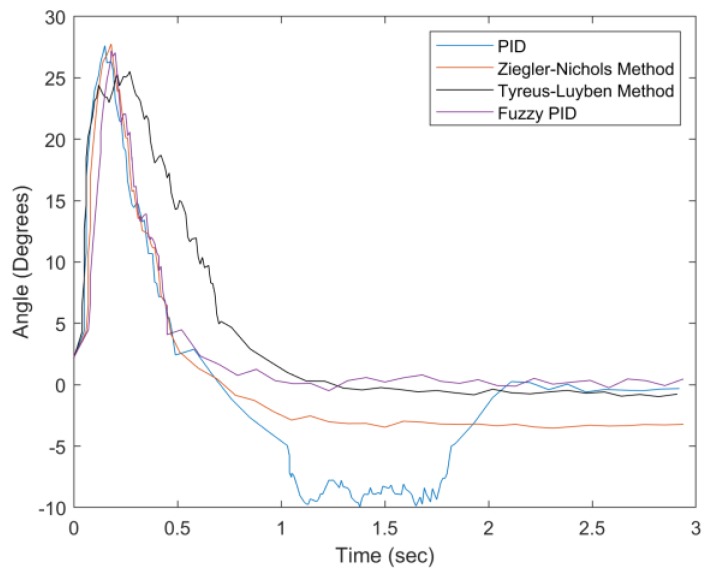
Tilt angle is approximately 25 degrees.

In the second set of experiments with a 250 g weight, the system performance was evaluated by varying the tilt angle from 20° to 30°. Given the tilt of 20°, Figure 34 shows that the Tyreus–Luyben method has the largest values of settling time, percentage undershoot, and steady-state error among the four test methods. Similarly, considering the tilt angel of 25°, Figure 35 shows that the proposed fuzzy PID method performs well in various weight conditions in terms of settling time, maximum overshoot, and steady-state error. Observe that the Ziegler–Nichols method has the largest steady-state error, and the PID method leads to a large percentage undershoot in the balance procedure. Moreover, although the Tyreus–Luyben method has a small steady-state error, it is time consuming to keep the system stable.

**Figure 36:**
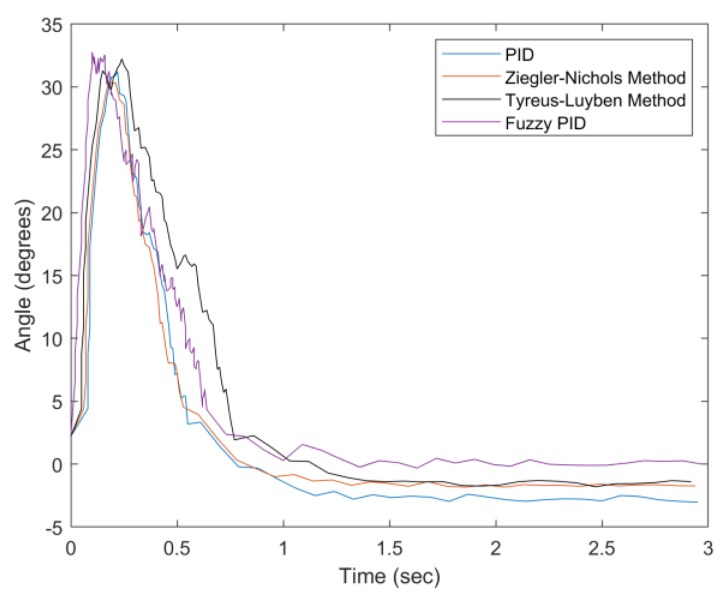
Tilt angle is approximately 30 degrees.

For the system with the tilt angle of 30°, among the four methods, the proposed method has the smallest steady-state error, which is compatible with respect to the Ziegler–Nichols method in terms of the settling time (Figure 36). It can be found that the convergence curve of the Tyreus–Luyben method is similar to that of the Ziegler–Nichols controller, due to the large parameter settings in the integral term, which speeds up the error accumulation to reduce the steady-state time. Notice that given a 250 g weight and varying the tilt angle from 20° to 30°, the proposed scheme has the smallest steady-state error and has no undershoot conditions, which indicates that the proposed system can be applied to facilitate patient’s mobility and ensure the patient’s safety. The experimental results are shown in Table 9 and Table 10. Observe that the proposed fuzzy PID method performs better than the other examined methods in various angle conditions in terms of settling time, percentage undershoot, and steady-state error.

## 5. Conclusions

This paper has proposed and implemented a control system for balancing a piggyback intravenous drip frame. The key features of the proposed system are as follows. (1) The device can be moved with the human body, (2) the system can cooperate with shoulder activities and neck activities, and (3) the control mechanism can adjust the tilting angle of the frame to automatically restore the angle. The performance of the proposed system is compared with those of the traditional PID controller, the Ziegler–Nichols method, and the Tyreus–Luyben method. The proposed fuzzy PID method performs better than the other examined methods in various weight and angle conditions in terms of settling time, percentage undershoot, and steady-state error. This work aims to contribute to the increased mobility and higher quality of life for the patient. Although the proposed system is promising, for future work, we intend to develop improved algorithms, such as the sensing information processing, balance control techniques, and improve designs for the vest, in order to create a reliable and balanced intravenous drip frame.

## Founding

This research was funded by the Ministry of Science and Technology of Taiwan under grant number MOST-108-2221-E-005-011, and by the “Innovation and Development Center of Sustainable Agriculture” from The Featured Areas Research Center Program within the framework of the Higher Education Sprout Project by the Ministry of Education (MOE) in Taiwan.

## Figures and Tables

**Table 1:** Comparison of drip frame technologies.

Type	Advantages	Disadvantages
Fixed drip frame, including pulleys	Moderate convenience of movement	Lack of agility and convenience of movement
A shoulder-mounted drip frame (e.g., Taiwan Patents M404714 [14] and M360702 [15], EZPole [16])	Convenience of movement	The drip frame may tilt with the user, which reduces the height of the infusion tube. Increase the risk of blood return.
The proposed system (balanced control)	Correct the drip frame position when a user tilts forward or backward. Avoid the intravenous drip’s blood flowing back problem.	Equipped with a control system

**Table 2:** Controller parameters for the closed loop Ziegler–Nichols method.

**Controller**	Kp	τ_i_	τ_d_
**P**	0.5Ku	-	-
**PI**	0.45Ku	Pu/1.2	-
**PID**	0.6Ku	Pu/2	Pu/8

**Table 3:** Controller parameters for the Tyreus–Luyben method.

**Controller**	Kp	τ_i_	τ_d_
**PI**	Ku/3.2	2.2Pu	-
**PID**	Ku/3.2	2.2Pu	Pu/6.3

**Table 4:** A fuzzy rule base.

Weight	Angle		
	Small	Medium	Large
**Light**	Kp = P Ki = S	Kp = P Ki = M	Kp = P Ki = L
**Medium**	Kp = PS Ki = S	Kp = PS Ki = M	Kp = PS Ki = L
**Heavy**	Kp = PM Ki = S	Kp = PM Ki = M	Kp = PM Ki = L
**Very heavy**	Kp = PL Ki = S	Kp = PL Ki = M	Kp = PL Ki = L

**Table 5:** Motor characteristics.

Nominal Voltage (V)	24
Rated Power (W)	10
No Load Speed (RPM)	15
Rated Speed (RPM)	13
Rated Torque (Kg*cm)	13
Stall Torque (Kg*cm)	78
Weight (g)	215

**Table 6:** System parameter settings.

**Sample Time Interval (sec)**	0.003
**System Dead Zone (degree)**	5
**R**	2
**Ku**	9
**Pu**	0.5

**Table 7:** Comparison of Experiment I. PU: percentage undershoot, ST: settling time.

Weight	100 g		150 g		200 g	
Method	PU	ST	PU	ST	PU	ST
**PID Controller**	9.5%	0.55	9.4%	0.46	33.1%	1.92
**Ziegler–Nichols**	11.5%	0.47	9.3%	0.42	9.4%	0.42
**Tyreus–Luyben**	--	0.73	2.0%	1.02	--	0.66
**Fuzzy PID**	5.7%	0.5	1.0%	0.54	0.2%	0.46

**Table 8:** Steady-state error in Experiment I.

	Weight	0 g	100 g	150 g	200 g
Method					
**PID Controller**		2.94°	2.36°	2.12°	1.28°
**Ziegler–Nichols**		1.33°	2.99°	1.77°	2.84°
**Tyreus–Luyben**		0.32°	1.11°	0.95°	0.6°
**Fuzzy PID**		0.57°	0.53°	0.71°	0.11°

**Table 9:** Comparison of Experiment II.

Angle	20°		25°		30°	
Method	PU	ST	PU	ST	PU	ST
**PID Controller**	8.9%	0.5	38.2%	1.83	6.1%	0.55
**Ziegler–Nichols**	6.9%	0.49	10.3%	0.47	5.5%	0.53
**Tyreus–Luyben**	13.4%	1.01	1.6%	0.76	4.4%	0.75
**Fuzzy PID**	--	0.46	--	0.45	--	0.64

**Table 10:** Steady-state error in Experiment II.

	Angle	20°	25°	30°
Method				
**PID Controller**		2.06°	0.24°	1.47°
**Ziegler–Nichols**		1.41°	3.25°	1.63°
**Tyreus–Luyben**		2.60°	0..51°	1.41°
**Fuzzy PID**		0.53°	0.23°	0.21°
